# Perianal Basal Cell Carcinoma—A Systematic Review and Meta-Analysis of Real-World Data

**DOI:** 10.3390/diagnostics13091650

**Published:** 2023-05-08

**Authors:** Tzong-Yun Tsai, Chun-Kai Liao, Bang-Yan Zhang, Yen-Lin Huang, Wen-Sy Tsai, Jeng-Fu You, Chien-Yuh Yeh, Pao-Shiu Hsieh

**Affiliations:** 1 Division of Colon and Rectal Surgery, Department of Surgery, Chang Gung Memorial Hospital at Linkou, Taoyuan City 33305, Taiwan; 2 College of Medicine, Chang Gung University, Taoyuan City 33305, Taiwan; 3 Department of Otolaryngology–Head and Neck Surgery, New Taipei Municipal Tucheng Hospital (Built and Operated by Chang Gung Medical Foundation), New Taipei City 23652, Taiwan; 4 Department of Anatomic Pathology, Chang Gung Memorial Hospital at Linkou, Taoyuan City 33305, Taiwan; 5 School of Medicine, National Tsing-Hua University, Hsinchu 300044, Taiwan; 6 Institute of Stem Cell and Translational Cancer Research, Chang Gung Memorial Hospital at Linkou, Taoyuan City 33305, Taiwan

**Keywords:** perianal basal cell carcinoma, diagnosis, anal malignancy, systematic review, oncologic outcome

## Abstract

(1) Background: BCC is a sporadic disease that develops in areas of the skin not exposed to the sun. Perianal BCC, which occurs in the anorectal region, accounts for less than 0.2% of all BCC cases. There have been only a few reported cases of the disease, with fewer than 200 cases reported in total. Given the diagnostic challenges and potential for misdiagnosis, we conducted a systematic review of perianal basal cell carcinoma using real-world data to provide comprehensive and detailed information on the disease. (2) Methods: The study was reported based on the Preferred Reporting Items for Systematic Reviews and Meta-Analyses (PRISMA) guidelines, 2020. Patients’ clinical pathologic features, tumor characteristics, treatment modalities, and outcomes were presented. (3) Results: The results of 41 studies involving 140 patients were analyzed. The most common symptoms reported by patients at presentation were anorectal bleeding, pain, and pruritus. Ulceration was the most frequently observed tumor characteristic. The majority of patients underwent local excision as their primary treatment, with only eight patients experiencing a recurrence. Our analysis did not reveal any statistically significant differences in the outcomes of different treatment modalities. (4) Conclusions: Identifying perianal BCC poses a significant challenge as it closely resembles other anal diseases, thereby making it difficult to differentiate between the different conditions. However, a wide local excision with clear margins is considered an effective treatment option for most patients. Alternative treatments, such as radiotherapy, may be recommended for patients who are unable to undergo surgery.

## 1. Introduction

In the non-melanoma skin cancer population, more than 50% of cases are attributed to basal cell carcinoma (BCC) [1]. BCCs typically arise in areas that are frequently exposed to the sun, such as the head or neck, and ultraviolet radiation is a major contributing factor. It is predominantly linked to the elderly population and is typically a slow-growing, localized disease. Advanced age and the male gender are considered independent risk factors for BCCs [2,3,4]. BCCs seldom metastasize, and the mortality rate is extremely low (<0.1) [5]. Nonetheless, the incidence of BCCs has been rising over the past few decades [4,6]. Thus, prevention of the disease holds significant importance.

BCC is a sporadic disease that develops in areas of the skin not exposed to the sun. Perianal BCC, which occurs in the anorectal region, accounts for less than 0.2% of all BCC cases. BCCs are staged based on tumor size and invasion, and early detection can lead to better patient outcomes [7]. 

Squamous cell carcinoma (SCC) is the most common type of anal cancer, while perianal BCC is the rarest among all anorectal malignancies [8]. However, there is a variant of SCC known as basaloid squamous cell carcinoma, which shares overlapping histological features with BCC, but is typically found in the anal canal and has a higher risk of distant metastasis compared to BCC [9]. The prognosis for basaloid carcinoma is worse than that for BCC. Perianal BCC arises from perianal lesions and tends to be a regional disease. SCC is usually treated with definitive concurrent chemoradiation therapy, and BCC is typically treated with local excision. Therefore, it is important to differentiate between these two types of cancer, and knowledge of the characteristics of perianal BCC can aid in a differential diagnosis.

Various treatment modalities have been reported for treating perianal BCC, including wide local excision, Mohs microscopic surgery, or radiotherapy. While topical imiquimod has not been used to treat perianal BCC, it has been shown to be effective in treating superficial BCCs [10]. For most patients, a standard excision with a negative margin is recommended to remove the cancer, while radiotherapy is used for patients who are not able to undergo surgery. Systemic treatments, such as inhibitors of the Hedgehog signaling pathway, are recommended for treating patients with recurrences at distant sites [11]. A review and analysis of the different treatment modalities for the disease can provide physicians with insight into the most effective treatment methods.

Perianal BCC can resemble benign conditions, such as hemorrhoids, fistulas, fissures, or infections, making it crucial for surgeons to consider the possibility of malignancy before surgery. However, due to the rarity of perianal BCCs, there have been only a few reported cases of the disease, with fewer than 200 cases reported in total. Retrospective studies on the disease are also limited in their coverage of important tumor characteristics, such as morphology, location, symptoms, or outcomes.

Given the diagnostic challenges and potential for misdiagnosis, we conducted a systematic review of perianal basal cell carcinoma using real-world data to provide comprehensive and detailed information about the disease. Our review covers the clinical and pathological features of patients, tumor characteristics, treatment modalities, and disease outcomes. We aimed to review both past and current research related to perianal basal cell carcinoma in a systematic manner.

## 2. Materials and Methods

The study is reported based on the Preferred Reporting Items for Systematic Reviews and Meta-Analyses (PRISMA) guidelines, 2020. The protocol is registered in PROSPERO (ID: CRD42022378097). 

### 2.1. Search Strategy and Study Selection

A comprehensive search was carried out systematically through the Embase, Medline, and PubMed databases, and covered a time period from the earliest publications in those databases up to December 2022. The search was carried out by two independent reviewers (T.Y. Tsai and C.K. Liao). The search strategy is presented in Appendix A. The terms “perianal”, “anus”, “basal cell carcinoma”, and “basal cell epithelioma” were included. In order to gather as much information as possible and to include as many cases of perianal BCC as possible, we searched for all papers or articles related to perianal BCC, including case reports, retrospective studies, letters to the editor, conference abstracts, and image challenges. The bibliographies of the included trials and related review articles were manually reviewed for potentially missing additional studies.

The study selection was made using Endnote software. Duplicate articles were identified and removed. Titles and abstracts that were irrelevant to our study were excluded. We then sought out the full texts of the remaining articles. Once the full texts were retrieved, they were screened in detail. The studies were removed if they met the following exclusion criteria: (1) the article was not written in English; (2) the study was not relevant to our subject; (3) the article dealt with other anal diseases, such as basosquamous cell carcinoma or other anal cancers; (4) the article was about basal cell carcinoma in other anastomotic sites; (5) the articles were reviews; or (6) the articles contained duplicated data. 

### 2.2. Data Extraction

All of the retrieved articles were reviewed by two independent reviewers, and the data were extracted from each article based on a predetermined format. For each report or study, the following data were extracted: age, gender, symptoms when diagnosed with BCC, any other BCC lesions, tumor morphology, tumor size, tumor location (which aspect of the anus, such as the anterior, posterior, right anterior, right lateral, right posterior, left anterior, left lateral, or left posterior), sphincter invasion, image studies, whether a biopsy was performed before surgery, and tumor staging according to the eighth edition of the American Joint Committee on Cancer (AJCC) Cancer Staging Manual [12]. Treatment modalities, including surgery, topical treatments, or radiotherapy, were recorded. Details on the surgical margins were collected, as were the outcome measures, including follow-up time, recurrence, and mortality. 

### 2.3. Quality Assessment

The CARE case report, which contains 13 guidelines and 30 subitems, was used for the quality assessment (QA) of the selected case reports, letters to the editor, image challenges, or conference abstracts. Each item was rated either yes or no. A final QA score was calculated from the sum of the subitems/all items of each study, and the result was presented as a percentage of these ratings. 

We used the STROBE checklist to assess the quality of the retrospective studies, which included 22 items rated as either yes or no. The final quality score was then presented as a percentage. The QA was carried out by two independent reviewers (T.Y. Tsai and C.K. Liao). 

### 2.4. Statistical Analysis

Since there were only a limited number of cases, studies that had missing data were still included in the Results section to prevent selection bias. Since there were only a few cases, we collected individual-level data from each study. In order to calculate the percentages for patients’ clinicopathologic characteristics and tumor characteristics, both the entire dataset and valid denominators (the details are found in the text) were used.

The variables that are categorical, such as gender, clinical presentation at diagnosis, and tumor characteristics, are presented as numbers and percentages, while continuous variables, such as age and follow-up time, are expressed as the mean and standard deviation (95% confidence intervals). 

As a comparison of the different treatments, we used the chi-square test, and a significance level of 0.05 was used to indicate the significant differences. We were unable to calculate the survival data for all individuals due to a limited amount of follow-up time.

A meta-analysis of the multiple local excisions, recurrences after local excisions, stoma rates, and abdominal-perineal resection (APR) rates was conducted using OpenMeta[Analyst]. Three retrospective studies were used to perform a meta-analysis of the results of the local excisions compared with other treatment modalities. Data were pooled, and the mean rates were weighted with a 95% confidence interval (CI). Heterogeneity was presented as I^2^ and considered high if I^2^ > 75%. A significant heterogeneity was indicated if *p* < 0.1. We used the binary random effect method for the pooling of the data. Due to the small sample size, the DerSimonian–Laird procedure was used for the random effect model. The statistics were carried out using SPSS 25 and OpenMeta [Analyst].

## 3. Results

### 3.1. Study Selection and Quality Assessment

**Figure 1:**
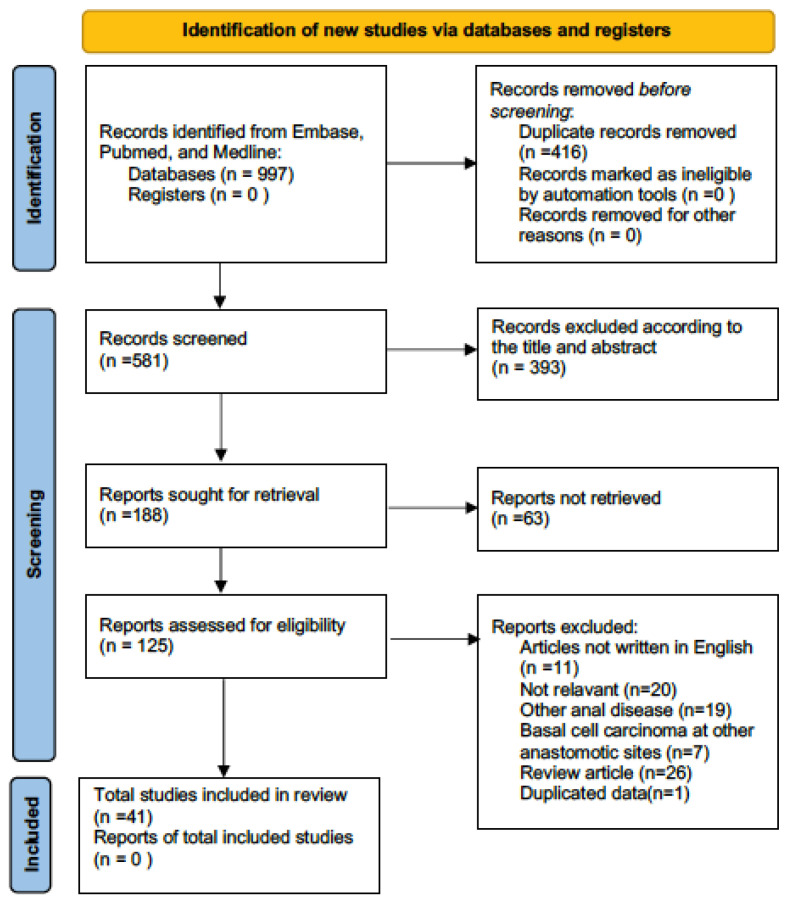
PRISMA flowchart.

On the basis of the online databases and manual searches, we identified 997 studies, of which 416 were duplicate records, so they were eliminated using both Endnote and a manual approach. Based on the reviews of the titles and abstracts of the remaining articles, 393 of them were removed due to their irrelevant subjects. The full-texts of a total of 188 articles were sought for retrieval, but the full-texts of only 125 articles were obtained. Based on a thorough assessment of the full texts of the remaining 125 articles, 84 were excluded as a consequence of the exclusion criteria: (1) eleven articles were not written in English; (2) twenty studies were not relevant to our subject; (3) nineteen articles dealt with other anal diseases, such as basaloid squamous cell carcinoma, other anal cancers, or other anal diseases; (4) seven articles dealt with basal cell carcinoma found in other anastomotic sites (*n* = 7); (5) twenty-six articles were reviews; and (6) one article contained duplicated data (*n* = 1). Finally, 41 studies that were eligible for inclusion were included in our review. Figure 1 shows the PRISMA flowchart, which is an overview of all of the processes involved in our selection.

There were 41 studies chosen for inclusion, including 30 case reports, four retrospective studies, four letters to the editor, two conference abstracts, and one image challenge. In the included studies, the data pertaining to a total of 140 patients with perianal BCC were subjected to analysis (Table 1). 

A quality assessment of the studies showed scores ranging from 23% to 90%. Over half of them had quality scores over 50%. Three retrospectives had quality scores over 50%. Overall, the quality of these studies was considered acceptable.

### 3.2. Clinical Features of the Patients

Out of the 140 patients included in this review, the ages of 16 patients were not reported. The mean age of the other 124 patients was 68.8 years (ranging from 33 to 93 years). There were more males (*n* = 86, 61.4%) than females (*n* = 54, 38.6%).

The symptoms before the diagnosis of perianal BCC were reported in 70 patients. Among these patients, the majority had anorectal bleeding (*n* = 24, 34.3%), pain (*n* = 21, 30%), and pruritus (*n* = 13, 18.6%) at the time of diagnosis. Nine patients (12.9%) did not have any discomfort when diagnosed.

Nineteen patients (13.6%) had previously experienced BCCs at other anatomic locations (such as the head and neck, trunk, or extremities, etc.) (see Table 2). Six patients (4.3%) had previous anal conditions or diseases, such as hemorrhoids (*n* = 2), anal fistulas (*n* = 2), anal trauma (*n* = 1), and anal fissures (*n* = 1). Two patients had a history of irradiation.

### 3.3. Characteristics of Perianal BCC

Out of all of the cases analyzed, 42 included descriptions of tumor morphology. Among these cases, most tumors were observed to have ulcerations (*n =* 28, 49.1%), raised edges (*n =* 16, 28.0%), and hyper-/hypopigmentation (*n =* 13, 22.8%).

In terms of tumor size, 66 patients had available data for analysis, and the data concerning the individual tumor size were available for 37 patients. The mean tumor size (maximum dimension) reported by Liu et al. was 2.2 cm and was included in the calculation of the average size. Upon combining the available data, the average tumor size (maximum dimension) was found to be 3.2 cm. Furthermore, Nielsen et al. reported tumor sizes in the following categories: tumors < 3 cm (*n =* 19), 3–5 cm (*n =* 12), and 5–10 cm (*n =* 3), which were not included in the average calculation. For the 37 patients with reported individual tumor sizes, staging was determined according to the information in the eighth edition of the American Joint Committee on Cancer (AJCC) Cancer Staging Manual, which is based on tumor size and depth of invasion. Of these patients, 10 had stage I tumors (27.0%), 13 had stage II tumors (35.1%), and 14 had stage III tumors (27.0%). No lymph nodes or distant metastases were observed in any of the cases. Sphincter invasion was reported in four cases, while the remaining cases either had no sphincter invasion (*n =* 30) or did not report a sphincter invasion (*n =* 106).

When considering the location of the tumors, a total of 69 patients were identified. The majority of tumors were located on the left side of the perianal region (*n =* 26, 37.7%) or on the posterior aspect (*n =* 19, 27.5%) (Table 3).

### 3.4. Diagnosis of Perianal BCC

Perianal BCCs were diagnosed based on pathologic confirmation obtained from a biopsy or wide local excision. Among the cases reviewed, 17 were diagnosed with perianal BCCs based on pathologic confirmation prior to treatment with surgery or radiotherapy. 

Among all cases, four patients underwent MRI before treatment. Of the four patients who underwent MR imaging, three were found to have tumors that had invaded the external sphincter. However, in the four retrospective studies included in the review, there was no information regarding whether the patients had a biopsy or MRI prior to treatment.

### 3.5. Treatment and Outcomes

The follow-up period for all cases ranged from 0.3 to 214 months from the date of treatment. The mean follow-up time for 37 case reports was 16.7 months (ranging from 0.3 to 54 months). Table 4 displays the follow-up period, treatment, and outcomes of four retrospective studies. 

One hundred and twenty-five patients with different treatment modalities are presented in Table 5. There were 22 mortalities and eight recurrences during the follow-up period. None of the patients died from perianal BCC. Most patients underwent local excision only (*n =* 102, 82.4%). Among the patients who underwent local excision, eight patients (7.8%) needed salvage therapy due to recurrences, which included repeated local excision (*n =* 5), radiotherapy (*n =* 2), or APR (*n =* 1). Five patients who were not able to undergo surgery were given radiotherapy only. Four patients underwent local excision along with adjuvant radiotherapy with no recurrence. Three patients had Mohs microscopic surgery without recurrence. Among all patients, APR was performed on six patients. 

**Figure 2:**
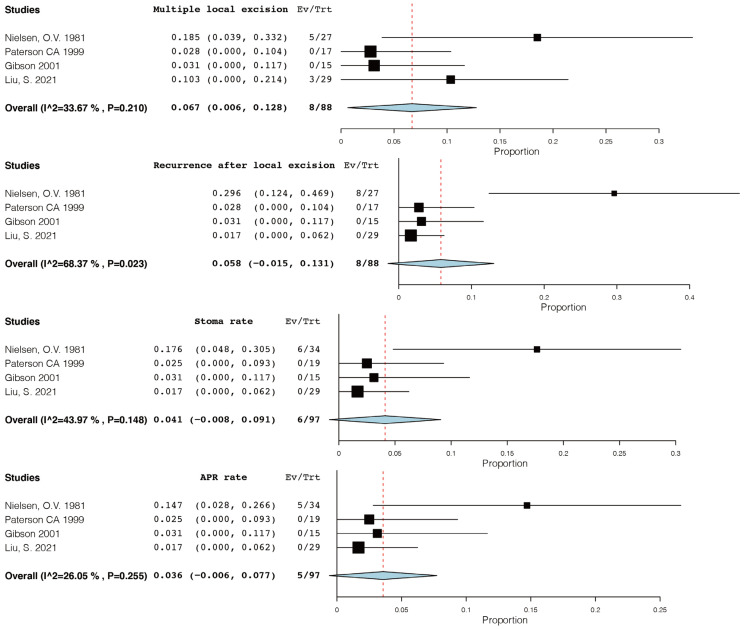
Forest plot of the multiple excision rates, recurrence rates after local excision, stoma rates, and APR rates of four retrospective studies [7,50,51,52].

The forest plots of four retrospective studies are presented in Figure 2. Among the patients who underwent local excision, a total of eight (9.1%) patients required multiple local excisions or re-excisions due to positive margins or recurrences (95% CI, 0.6–12.8, I^2^ = 33.67%) and eight (9.1%) patients had a recurrence after local excision (95% CI, −1.5–13.1, I^2^ = 68.37%). Six (6.2%) patients eventually had a stoma constructed (95% CI, −0.8–9.1, I^2^ = 43.97%), and five (5.2%) patients had APR (95% CI, −0.6–7.7, I^2^ = 26.05%). 

Among all of those who underwent excision, surgical margins were reported in 43 cases (37.1%). In the first instance, there were four cases involving margins that required multiple excisions. Lie et al. reported that their average margin for 29 cases was 1.08 cm. The margins of the other cases ranged from 1 mm to 1 cm. There were no recurrences in these cases.

In the case reports, four patients underwent stoma construction: two of whom received radiotherapy after the procedure, one underwent a proctocolectomy with end ileostomy due to chronic diarrhea, and one had planned radiotherapy but passed away nine days later due to myocardial infarction. Additionally, treatment with steroids for perianal lesions was unsuccessful in two patients, and they ultimately required excision after the treatment failed. 

The statistical analysis shows that there are no significant differences in treatment outcomes between the different modalities (Table 5). We conducted a comparison of the local excisions with other treatment options across three retrospective studies, but excluded Gibson’s study because it did not provide sufficient information. Notably, Gibson’s study reported no recurrence of perianal BCC. 

**Figure 3:**
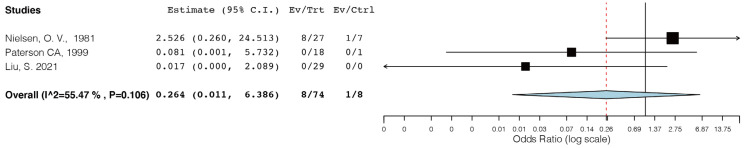
Comparison of the local excisions with other treatment modalities [50,51,52].

The Forest plots show that local excisions did not differ significantly from other treatments (95% CI, 0.01–6.4, I^2^ = 55.47%) (Figure 3). However, due to the limited number of cases, the results of the analysis were not statistically significant. We were unable to calculate the survival rate due to a lack of follow-up data on individual patients.

## 4. Discussion

The present study provides a comprehensive review of perianal BCC, representing, to our knowledge, the first such analysis. The aim was to provide a detailed understanding of the disease, including patient symptoms, tumor characteristics, treatment, diagnosis, and outcomes. Perianal BCC is typically a slow-growing regional disease and may even be asymptomatic. Most patients in the study underwent local excision, and only a few experienced recurrences after treatment. A meta-analysis of three retrospective studies showed no significant differences in outcomes between local excision and other treatments. However, limited data on recurrence rates from only one retrospective study makes it difficult to draw definitive conclusions. As such, this study presents valuable real-world data on the management of perianal BCC to date. 

### 4.1. Epidemiology of Perianal BCCs

More than half of all keratinocyte carcinomas are basal cell carcinomas (BCCs), and the remainder are squamous cell carcinomas (SCCs) [1]. High densities of both BCCs and SCCs are observed on sun-exposed areas, such as the head and neck, where sun exposure is the main etiological factor. However, a study of 5150 keratinocyte tumors found that on the least exposed sites, such as the back and/or buttocks, BCCs occurred at a relative tumor density that was more than eight-fold higher than for SCCs [5]. In contrast, anal squamous cell carcinomas comprise 80% of all anal cancers [53], while anal basal cell carcinomas only comprise 0.2% of all anorectal cancers [9,51]. The difference is that anal SCCs correlate highly with HPV/HIV infection, but not with UV light exposure. 

Our study found that most patients with perianal BCC were of old age, with a mean age of around 70 years. Age is an independent risk factor for basal cell carcinoma, with the incidence rate doubling from 40 to 70 years old [2,3,4]. In contrast, most anal SCCs occur in patients aged 50 years or older [54].

Our review also showed that there were more male patients than female patients with perianal BCC. These data are consistent with previous studies on all basal cell carcinomas, which found that men have a higher rate of BCC [2,3,4]. In contrast, anal SCCs occur more frequently in female patients. 

Patients with their first BCC have a higher rate of developing a second BCC, with approximately one-third of patients experiencing this. Our data are compatible with this finding, as 19 out of 70 patients had a previous BCC history [55]. 

According to our review, there were no distant metastases at the time of the diagnosis of perianal BCCs. Most BCCs are localized and regional diseases, with metastasis rates ranging from 0.0028% to 0.55%. As a result, the mortality rate is very low (<0.1) [5]. In comparison, anal SCCs, which are mostly localized and regional diseases, have an 8% rate of distant metastasis and a 0.16% mortality rate [54]. 

### 4.2. Etiology of Perianal BCCs

The primary cause of basal cell carcinoma is exposure to ultraviolet radiation [56]. A previous study reviewed the molecular pathogenesis of this cancer, including the inappropriate activation of the Hedgehog (HH) signaling pathway and mutation in the p53 tumor-suppressor gene. Ultraviolet radiation exposure can lead to gene mutations that contribute to the development of basal cell carcinoma [2]. 

Basal cell carcinoma may be caused by various types of exposure, besides ultraviolet radiation, such as exposure to arsenic, ionizing radiation, coal tar, smoking, oral methoxsalen, or trauma. In addition, patients with compromised immune systems are at increased risk of developing the disease [2,7,57].

Gibson et al. reviewed 51 cases of BCC in the perianal and genital areas, and out of these, 15 were perianal. The study showed that chronic skin irritation, trauma, or a past history of radiation of the perineum might also be reasons for BCCs in the perianal area [7]. According to the review we conducted, two patients had hemorrhoids, two patients had an anal fistula, two patients had a history of irradiation, one patient had anal trauma, and one patient had an anal fissure prior to the diagnosis of perianal BCC.

Our review also revealed that 27.1% of patients had a history of BCCs at another site, and patients with a history of BCCs at other sites or other skin cancers may also have an increased risk of developing BCC in the perianal area. The National Cancer Institute recommends conducting a whole-body skin examination every six to twelve months for the first five years after a diagnosis of BCC. Following this period, annual inspections should be performed [11].

### 4.3. Characteristics and Diagnosis of Perianal BCC

**Figure 4:**
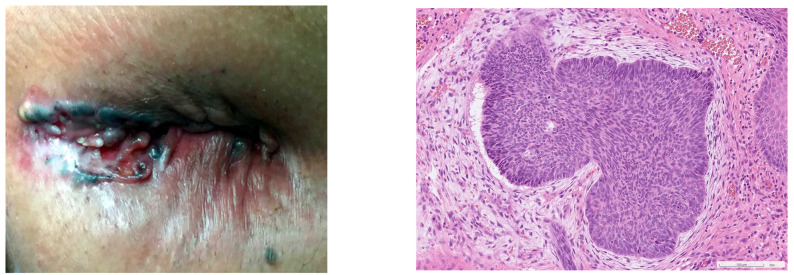
An image of perianal basal cell carcinoma located at the posterior of the anus with a central ulceration, raised edges, and hyperpigmentation. A pearl-like nodular lesion is also visible. The histological image is shown on the right. There is a slit-like space between the tumor nest and the surrounding desmoplastic stroma. The tumor cells have peripheral nuclear palisading and occasional mitotic figures. The diagnosis is a nodular-type BCC.

BCCs can display varying morphologies: the typical BCC presents a pearly, nodular, flesh-colored papule with telangiectasia and a smooth margin. Infiltrative BCCs may feature a scaly surface with an ill-defined margin [58]. Unlike BCCs at other anastomotic sites, most perianal BCCs present as ulcerated lesions. This finding is consistent with a prior study that reported that 29.4% of 51 patients with perineal BCC had ulcerated lesions [7]. A typical description of perianal BCC would be a “central erythematous ulceration with a hard-raised edge with or without a hyperpigmented margin” (Figure 4). It is critical to differentiate perianal BCC from other diseases that cause anal ulcerations, such as anal fissures, fistulas, or infectious diseases, including chancroid, HSV infection, HIV infection, or diseases of an undetermined nature [59]. 

Regarding anal malignancies, squamous cell carcinoma is the most common type and can present as an ulcerative lesion. One notable difference between basal cell carcinoma and anal malignancies is that BCC primarily affects the perianal area without invading the anal canal or rectal mucosa. In contrast, anal SCCs are typically found within the anal canal. A definitive diagnosis requires obtaining pathologic confirmation. A subtype of squamous cell carcinoma, known as basaloid carcinoma, shares histological features with basal cell carcinoma, but has a higher risk of distant metastasis and a worse prognosis. To distinguish between these two subtypes, immunohistochemical markers, such as diffuse Ber-EP4 and BCL2 staining are typical of basal cell carcinoma, while diffuse CDKN2A and SOX2 expressions are almost exclusively associated with basaloid squamous cell carcinoma. The presence of an adjacent precursor lesion supports the diagnosis of basaloid squamous cell carcinoma over basal cell carcinoma [9].

Our review revealed that BCC situated in the perianal area tends to be diagnosed at a larger size than those located on the head and neck. Typically, tumors on the head and neck are first diagnosed with a diameter of one to two centimeters. However, our data indicate that patients with perianal BCCs are often diagnosed with tumors that have an average size of three centimeters in diameter [60]. The size of the BCC is a crucial factor in determining the appropriate treatment and the outcome of the treatment. Therefore, early detection and proper management of BCC are crucial [11]. 

Anal pain, bleeding, and pruritus are commonly observed as the initial symptoms of perianal BCC. In a single reported case, recurrent pruritus ani was the presenting symptom, and despite unsuccessful attempts with topical agents, local excision was eventually performed, leading to the diagnosis of BCC [20]. 

Based on the review, most perianal BCC lesions were found in the left and posterior areas of the anus. It has been suggested that the lower perfusion in the posterior aspect of the anus may contribute to the higher incidence of anal fissures in this area, but no correlation has been established between anal fissures and BCC. The reason for the left-side predominance of perianal BCCs is unknown.

Among the cases reviewed, only four patients underwent MRI to assess the extent of the lesion. While MRI is practical for the local staging of anal and rectal carcinomas, its use for superficial and regional diseases, such as BCC, is not recommended. The diagnosis of BCC in uncommon locations can be challenging. Therefore, this systematic review provides a comprehensive overview and describes the clinicopathologic features of the disease that may be useful for further research and clinical practice.

### 4.4. Treatment and Outcomes of Perianal BCC

As per the guidelines of the National Comprehensive Cancer Network (NCCN), basal cell carcinoma (BCC) situated in the anogenital region is considered a high-risk BCC, regardless of its size [11]. For patients who are eligible for surgery, the recommended initial treatment for basal cell carcinoma (BCC) is a standard excision with a negative margin. In cases of high-risk primary BCCs, a surgical margin of 4–5 mm is suggested. For recurrent lesions, a surgical margin of 6 mm or Mohs microscopic surgery is recommended [61].

Based on the available literature, it is suggested that multiple excisions may be required to achieve a negative margin in the treatment of perianal BCC. Previous reports have shown a relatively high rate of recurrence for surgical excision of facial BCCs, with a 10-year cumulative probability of 12.2% [62]. However, in our report, only one study by Nielsen O. V. et al. reported a recurrence after local excision, while most patients in other studies did not experience a recurrence during the follow-up period.

If local recurrences were detected after initial treatment, our preferred treatment options would be re-excision or radiotherapy. In a study by Nielsen et al., eight recurrent patients were treated, of whom five were treated with re-excision, two with radiotherapy, and one with APR. Hedgehog pathway inhibitors, such as vismodegib or sonidegib, can be utilized for patients with a nodal or distant recurrence. Systemic therapy was not used for perianal BCC patients in our report. Only one inguinal node recurrence was observed, which was successfully treated with radiotherapy [11].

For patients who are not suitable for surgery, definitive radiotherapy is the preferred treatment option. The recurrence rate was found to be 7.4% [63]. Adjuvant radiotherapy has not been shown to be effective in the treatment of perianal BCC. Its use is controversial and should only be considered in the case of extensive disease [8]. As part of our study, four patients received adjuvant radiotherapy after excision and five patients received RT alone, and none of these patients experienced recurrences. 

Although no report has used it to treat perianal BCCs, topical imiquimod also has a role in treating superficial BCCs. The inclusion of topical imiquimod in BCCs includes extensive or multiple tumors, cosmetically sensitive anatomic sites (face), a history of hypertrophic scarring and/or keloids, surgical risk factors, patient refusal to undergo a surgical procedure, or patients with a psychological condition [10]. Further investigation is needed for the use of topical imiquimod on perianal BCCs.

The limitations of this study are primarily due to the rarity of the disease, which means that a meta-analysis with a substantial outcome cannot be performed due to the small number of cases. Therefore, this study only offers real-world up-to-date data. Although we utilized four retrospective studies for our meta-analysis, we discovered that only one study conducted by Nielsen in 1981 reported a relatively high recurrent rate, stoma rate, and APR rate. This particular work is the oldest among the four retrospective studies, and the treatment methods may have varied over time. Furthermore, there is limited information about each case report and retrospective study. Nevertheless, we reviewed and presented as many cases as possible from the three databases and provided an overview of perianal BCCs.

## 5. Conclusions

Identifying perianal BCC presents a considerable challenge due to its resemblance to other anal diseases. However, a confirmed diagnosis warrants a wide local excision with clear margins, which remains the standard treatment for most patients. For patients who are not surgical candidates, alternative treatments, such as radiotherapy, can be administered. As perianal BCC is a rare disease with limited published cases, further investigation is required to improve our understanding of the disease, its clinical course, and optimal treatment approaches.

## Figures and Tables

**Table 1:** General data of the included studies.

Year	Author	Sample Size	Study Type	Gender	Age/Mean or Median (Range)	Quality Score
1949	Lott, B.D. [13]	1	Case report	F	62	50%
1954	Klippel [14]	1	Case report	M	65	50%
1955	Manheim, S.D. [15]	1	Case report	M	72	50%
1956	Case, T.C. [16]	1	Case report	F	72	63%
1958	Bunstock, W.H. [17]	1	Case report	M	93	40%
1958	Hanley, P.H. [18]	3	Case report	1M2F	72, 68, 65	53%
1958	Rosenberg and Rosen [19]	1	Case report	F	49	37%
1966	Turell, R. [20]	1	Case report	M	not reported	53%
1978	Kraus [21]	1	Case report	F	82	53%
1981	Baruchin [22]	1	Letter to the editor	F	64	23%
1992	Espana, A. [23]	1	Case report	F	72	47%
1992	Kyzer, S. [24]	1	Letter to the editor	F	33	37%
1995	Kort [25]	1	Case report	M	88	23%
1996	Alvarez-Cañas [26]	5	Case report	1M4F	62.8 (36–84)	53%
2001	Karim, R. [27]	1	Case report	M	83	53%
2002	Damin, D.C. [28]	1	Case report	F	77	47%
2003	Ruiz-de-Erenchun, R. [29]	1	Letter to the editor	M	90	33%
2004	Misago, N. [30]	1	Case report	F	88	53%
2010	Naidu, N. [31]	1	Case report	M	69	60%
2010	Shaikh, F.M. [32]	1	Image challenge	M	72	43%
2012	Kreuter, A. [33]	1	Case report	M	88	53%
2012	Lohana, P. [34]	1	Case report	M	58	53%
2012	Oliphant, T. [35]	1	Conference abstract	F	82	43%
2012	Yasar, S [36]	1	Case report	F	50	53%
2013	Kahn, S. [37]	1	Case report	F	62	53%
2013	Ng, K.-S. [38]	1	Case report	M	80	33%
2015	Bulur, I. [39]	1	Case report	M	34	60%
2015	Lee, H.S. [40]	1	Letter to the editor	M	83	50%
2016	Rivera-Chavarrí [41]	1	Case report	F	93	73%
2018	Carr, A.V. [42]	1	Case report	M	66	80%
2019	Aldana, P.C. [43]	1	Case report	M	89	53%
2019	Meeks, M. [44]	1	Case report	M	49	50%
2020	Hagen, E.R. [45]	1	Case report	M	67	57%
2020	Sharma, S. [46]	1	Conference abstract	F	73	43%
2021	Imbernon-Moya, A. [47]	1	Case report	F	68	43%
2022	Lim, M.G. [48]	1	Case report	F	60	70%
2022	Tung, J. [49]	1	Case report	M	69	90%
1981	Nielsen, O.V. [50]	34	Retrospective study	18M16F	68 (43–86)	64%
1999	Paterson, C.A. [51]	19	Retrospective study	15M4F	67(43–81)	64%
2001	Gibson [7]	15	Retrospective study	9M6F	73(45–100)	64%
2021	Liu, S. [52]	29	Retrospective study	23M6F	70 (43–90)	73%

**Table 2:** Clinical features of the patients.

	Mean
Age at diagnosis (*n =* 124)	68.8 ± 8.9
Gender (*n =* 140)	
Male	86 (61.4)
Female	54 (38.6)
Clinical presentation at diagnosis	Reported cases (% out of 70)
No symptoms	9 (12.9)
Pain	21 (30.0)
Bleeding	24 (24.3)
Pruritus	13 (18.6)
Change in bowel habits	9 (12.9)
Anal discharge	3 (4.3)
History of BCCs at other anastomotic sites	Reported cases (% out of 70)
Yes	19 (27.1)
No	51 (72.9)
History of other anal diseases	Reported cases (% out of 70)
Yes	6 (8.6)
No	64 (91.4)

**Table 3:** Characteristics of perianal basal cell carcinoma.

Tumor Morphology	*n* (% Out of 42)
Polypoid/Nodular	2 (4.8)
Pedunculated	1 (2.4)
Induration	6 (14.3)
Ulceration	28 (66.7)
Fungating	4 (9.5)
Raised edge	16 (38.1)
Hyper-/Hypopigmentation	13 (31.0)
Tumor size	*n* (% out of 37)
≤20 mm	10 (27.0)
>20 mm to ≤40 mm	14 (37.8)
>40 mm	13 (35.1)
Tumor stage	*n* (% out of 37)
Stage I	10 (27.0)
Stage II	13 (35.1)
Stage III	14 (37.8)
Stage IV	0 (0)
Tumor location	*n* (% of 69)
Anterior	5 (7.2)
Right anterior	3 (4.3)
Right	9 (13.0)
Right posterior	3 (4.3)
Posterior	19 (27.5)
Left posterior	1 (1.4)
Left	26 (37.7)
Left anterior	1 (1.4)
Circumferential	2 (2.9)
Sphincter invasion	*n* (% of 140)
Yes	4 (2.9)
No	30 (21.4)
Not reported	106 (75.7)

**Table 4:** Details of four retrospective studies.

Studies	Case Number	Treatment	Follow Up Times	Outcomes
Nielsen, O.V., 1981 [50]	34	Local excisions (*n =* 27) APR (*n =* 4) Colostomy (*n =* 1)	Not reported	Five-year OS 72.6%. Eight (23%) patients had a recurrence after local excision. One patient had an inguinal recurrence. Sixteen mortalities of other causes.
Paterson C.A., 1999 [51]	19	Local excisions (*n =* 17) Mohs microsurgery (*n =* 1) Electrodesiccation (*n =* 1)	72 months (2–214)	No recurrence. Four mortalities of other causes.
Gibson, 2001 [7]	51 (15 perianal, 36 genital)	Wide excision (*n =* 32) Electrodesiccation and curettage (*n =* 10), Mohs micrographic surgery (*n =* 8) Carbon dioxide laser (*n =* 1)	5 years or more	One recurrence of a superficial BCC of the vulva.
Liu, S., 2021 [52]	29	Local excisions (*n =* 29)	5.5 ± 4.6 years	No recurrence. No mortality.

**Table 5:** Treatments and outcomes for perianal BCC.

	*n*	Recurrence	*p* Value
Radiation therapy only	5	0	0.94
Radiation therapy followed by cryotherapy	1	0	
Electrodesiccation	1	0	
Local excision only	102	8	
Local excision plus adjuvant RT	4		
Radiation therapy followed by Mohs microscopic surgery	1	0	
Mohs microscopic surgery	3	0	
Radiation therapy followed by APR	1	0	
APR	5	0	
Others	2	0	

## Data Availability

The data presented in this study are available in this article.

## Appendix A. Search Strategy

Embase	#1 ‘basal cell carcinoma’/exp OR ‘basal cell carcinoma’
	#2 ‘perianal’ OR ‘anus’
	#3 ‘epithelioma’
	#4 ‘basal cell’
	#5 #1 AND #2
	#6 #2 AND #3 AND #4
	#7 #5 OR #6
Medline (Clarivate Analytics)	(basal cell) AND (carcinoma or epithelioma) AND (perianal or anus or anal)
PubMed	#1 ‘basal cell carcinoma’
	#2 ‘perianal’ OR ‘anus’
	#3 ‘epithelioma’
	#4 ‘basal cell’
	#5 #1 AND #2
	#6 #2 AND #3 AND #4
	#7 #5 OR #6
